# The Association between Provider Practice and Knowledge of ORS and Zinc Supplementation for the Treatment of Childhood Diarrhea in Bihar, Gujarat and Uttar Pradesh, India: A Multi-Site Cross-Sectional Study

**DOI:** 10.1371/journal.pone.0130845

**Published:** 2015-06-22

**Authors:** Laura M. Lamberti, Christa L. Fischer Walker, Sunita Taneja, Sarmila Mazumder, Robert E. Black

**Affiliations:** 1 Department of International Health, Johns Hopkins University Bloomberg School of Public Health, Baltimore, Maryland, United States of America; 2 Center for Health Research and Development, Society for Applied Studies, New Delhi, India; Johns Hopkins Bloomberg School of Public Health, UNITED STATES

## Abstract

**Introduction:**

Programs aimed at reducing the burden of diarrhea among children under-five in low-resource settings typically allocate resources to training community-level health workers, but studies have suggested that provider knowledge does not necessarily translate into adequate practice. A diarrhea management program implemented in Bihar, Gujarat and Uttar Pradesh, India trained private sector rural medical practitioners (RMPs) and public sector Accredited Social Health Activists (ASHAs) and Anganwadi workers (AWWs) in adequate treatment of childhood diarrhea with oral rehydration salts (ORS) and zinc. We used cross-sectional program evaluation data to determine the association between observed diarrhea treatment practices and reported knowledge of ORS and zinc among each provider cadre.

**Methods:**

We conducted principal components analysis on providers’ responses to diarrhea treatment questions in order to generate a novel scale assessing ORS/zinc knowledge. We subsequently regressed a binary indicator of whether ORS/zinc was prescribed during direct observation onto the resulting knowledge scores, controlling for other relevant knowledge predictors.

**Results:**

There was a positive association between ORS/zinc knowledge score and prescribing ORS and zinc to young children with diarrhea among private sector RMPs (aOR: 2.32; 95% CI: 1.29-4.17) and public sector ASHAs and AWWs (aOR 2.48; 95% CI: 1.90-3.24). Controlling for knowledge score, receipt of training in the preceding 6 months was a good predictor of adequate prescribing in the public but not the private sector. In the public sector, direct access to ORS and zinc supplies was also highly associated with prescribing.

**Conclusions:**

To enhance the management of childhood diarrhea in India, programmatic activities should center on increasing knowledge of ORS and zinc among public and private sector providers through biannual trainings but should also focus on ensuring sustained access to an adequate supply chain.

## Introduction

In India, approximately 140,451 deaths among children under-five were attributable to diarrhea in 2013 [1]. Although this figure has decreased substantially from 354,476 in 2000 [1], India continues to rank first on the list of childhood diarrheal deaths as more children die from diarrhea in India than in any other country in the world [2]. As of 2006, the diarrhea treatment guidelines issued by the Government of India and the Indian Academy of Pediatrics include reduced osmolarity oral rehydration salts (ORS) and 14 days of supplementation with 20 mg of zinc/day for children ≥ 6 months and 10 mg of zinc/day for children 2–5 months of age [3, 4].

National efforts to control childhood diarrhea in India have largely focused on increasing access to adequate treatment through public sector channels [5]. Providers at all levels of the public health system play a role in managing diarrhea among children under-five. Medical officers are essential to the treatment of severe cases seeking care at primary health centers (PHCs). Auxiliary nurse midwives and Accredited Social Health Activists (ASHAs) undertake child health activities in villages and are therefore vital to early detection and prompt treatment of diarrhea cases occurring in the community. Anganwadi workers (AWWs) are generally responsible for pre-school education and not typically allowed to dispense medicine, but state governments in Bihar, Gujarat and Uttar Pradesh (UP) have recently issued stipulations permitting AWWs to distribute ORS and zinc in an effort to leverage their position in the community among mothers of young children.

Despite national and state efforts encouraging utilization of public sector services for diarrhea treatment, about 80% of care-seeking for diarrhea takes place through the private sector [6, 7]. The Indian private health sector consists of formally qualified medical doctors, as well as informal providers referred to as rural medical practitioners (RMPs). The term RMP is loosely used to refer to several provider types, including those with government-recognized degrees in modern allopathic/traditional *‘Ayush’* (Ayurveda, Unani, Siddha, Homeopathy) medicine and those lacking formal training [7–9]. The majority of RMPs are unqualified and unregulated, falling under the latter category; however, their services fill a gap in rural communities isolated from the government health system [7, 9]. Consequently, RMPs and other private practitioners play a significant role in treating childhood diarrhea in India, but they typically prescribe injections, antibiotics and anti-diarrheal medications rather than ORS and zinc [7–9].

The success of childhood diarrhea management programs in India is thus largely dependent on the practices of community-level public and private sector providers, such as ASHAs, AWWs and RMPs. Diarrhea management programs often focus resources on training such providers in an effort to increase their knowledge of adequate childhood diarrhea treatment, including ORS and zinc. However, increasing provider knowledge is not sufficient to improve childhood diarrhea outcomes if improved knowledge does not translate into improved practice. For example, a study of mothers of children under-five in the Gambia reported that despite high levels of knowledge, utilization of ORS to treat diarrhea among young children was low [10]. Likewise, a pre- and post-educational intervention study among pharmacy workers in Vietnam found that high levels of reported knowledge were not necessarily correlated with adequate diarrhea prescribing practices during simulated patient consultations [11].

Research is therefore warranted to determine the role of provider knowledge on adequate diarrhea treatment in India in order to ascertain the relative importance of investments in provider training in the grand scheme of diarrhea management programs. Using data collected during the evaluation of diarrhea management programs in Bihar, Gujarat and UP, we aimed to assess the association between observed prescribing practices and reported knowledge of ORS and zinc among RMPs, ASHAs and AWWs.

## Methods

### Sampling and study design

The evaluation was designed as a cross-sectional, multi-site, observational study. The public sector provider arm of the evaluation was conducted from October-November 2011 in Bihar and from December 2011 –January 2012 in Gujarat; the private sector arm was carried out from June-July 2012 in UP. The provider assessment phase of the evaluation was intended to serve as a process evaluation to assess the early stages of program implementation and was thus timed to coincide with the completion of provider trainings in each state. Public sector training of ASHAs and AWWs consisted of distribution of written and pictorial educational materials and formal classes on diarrhea prevention and treatment. During these sessions, groups of ASHAs and AWWs received firsthand instruction and demonstrations on how to prepare and administer ORS and zinc and on how to relay this training to caregivers of children with diarrhea. ASHAs and AWWs were also made aware of the program’s supply chain model, which would allow them to obtain combination packs consisting of two ORS sachets and 14 zinc tablets at their required monthly ASHA/AWW meetings. In the event that ASHAs/AWWs finished their entire supply of ORS-zinc combination packs prior to the monthly meeting, they were instructed to still advise ORS and zinc treatment and to refer the child to the PHC where these products were likely to be in stock. Participation in the training sessions for ASHAs/AWWs was obligatory and lasted 1–2 days.

In the private sector, RMP training followed a profit-driven model and did not include formal classroom sessions. Pharmaceutical and NGO representatives were employed by the program implementers to identify RMP practices and conduct one-on-one visits, during which RMPs were shown a short video about adequate diarrhea treatment with ORS and zinc. These visits were typically short in duration to avoid frustrating RMPS by interrupting their patient consultations. Although adequate diarrhea treatment was discussed, the main goal of RMP visits was to explain the potential profits in zinc prescribing and the comparability of zinc profits to those earned through sales of non-routinely recommended drugs for childhood diarrhea, such as antibiotics and antidiarrheals.

During the provider assessments, we employed a multi-stage cluster sampling design in which the secondary sampling units were providers, and the primary sampling units were PHCs in the public sector and tehsils, a geographic unit, in the private sector. We used probability proportional to size (PPS) sampling to select the primary sampling units from sampling frames consisting of 236 PHCs in Gujarat, 69 PHCs in Bihar and 60 tehsils in UP.

We subsequently obtained sampling frames listing the ASHAs/AWWs employed at selected PHCs and the RMPs located within the area of selected tehsils. The public sector sampling frames were made available by PHC administrators and listed the names of approximately 6000 ASHAs and AWWs. The private sector sampling frames were acquired from program staff who identified and mapped RMPs prior to program implementation; these sampling frames listed the names of 40,900 RMPs. Using the public and private sector sampling frames, we randomly selected ASHAs, AWWs and RMPs for inclusion in the provider assessment.

The provider assessment included two parts: 1) an observation of the provider in consultation with one eligible diarrhea case and 2) a 30-minute interview. The observation was conducted first to prevent biasing the provider’s behavior with the content of the interview questions. Children eligible for observation were 2–59 months of age and suffering from a current, untreated diarrheal episode as defined by passage of at least three loose/watery stools in the previous 24 hours. In order to identify such cases during the public sector assessments, trained interviewers accompanied ASHAs and AWWs during their routine visits in the community and enrolled the first child meeting the eligibility criteria. During the private sector assessment, interviewers waited for a period of one working day at the selected RMP’s practice, and the first child seeking care and meeting the eligibility criteria was enrolled. Children were excluded if they had already received treatment outside the home for the current diarrheal episode and/or their caregiver did not verbally consent.

Once a consenting, eligible child was identified, interviewers observed the routine treatment practices of providers. Using an observation form specifically designed for this study, interviewers silently noted whether the provider asked the caregiver about diarrheal symptoms and episode duration. The interviewer also recorded any treatments recommended or provided during the consultation, as well as specific instructions on their usage (e.g. ORS preparation and zinc dosage). If a child eligible for observation was not identified during the ASHA/AWW’s routine visits or within 24-hours of waiting at the RMP practice, the interviewer skipped the observation and proceeded with the survey portion of the assessment. Following the observation, providers were interviewed in a private location. The interview lasted about 30 minutes and included questions on diarrhea management knowledge and practices and access to routinely available ORS and zinc supplies. A provider was considered to have access to ORS/zinc if he/she had the product in possession or was able to lead the interviewer to the location at which the product was stored.

For this analysis, we solely included data from providers completing both observations and interviews. We completed observations and interviews for 330 ASHAs and 330 AWWs in Bihar and Gujarat and for 97 RMPs in UP. Completion of RMP observations was impeded by lower-than-anticipated diarrhea care-seeking from RMP practices at the time of the study. Post-hoc power calculations completed in Stata 12.0 confirmed that at the alpha = 5% level, the achieved sample sizes granted 85% power to detect the proportion of RMPs ± 15% and the proportion of ASHAs/AWWs ± 8% that prescribed both ORS and zinc to observed children with diarrhea [12].

### Ethical permissions

We obtained informed written consent from all included providers; in the case of illiterate providers, a thumbprint was obtained in lieu of a signature in the presence of a literate witness from the community. Identifying information was not collected on children included in the observation portion of the assessment, so verbal, rather than written, assent was gathered from the primary caregivers of such children. During the consent process for both providers and caregivers, trained interviewers read aloud a script explaining the study and subsequently addressed questions and concerns. All consenting participants were provided with an information sheet and the phone number of a local researcher who was available for follow-up questions. These procedures were conducted in Hindi in Bihar and UP and in Gujarati in Gujarat. Ethical permissions for the study and approval of the consent procedures were granted by the Institutional Review Board (IRB) of the Johns Hopkins University Bloomberg School of Public Health in Baltimore, Maryland, USA and by the Ethical Review Committee (ERC) of the Society for Applied Studies in New Delhi, India.

### Construction of knowledge indexes

The main goal of our analysis was to determine whether provider knowledge of ORS and zinc was associated with prescribing practices during observed treatment consultations. The first step in achieving this aim was to construct a scale to quantify providers’ reported knowledge of ORS and zinc treatment for diarrhea among children 2–59 months of age. We used provider survey responses on zinc and ORS use, zinc dose and duration, and ORS preparation to generate a set of binary variables equal to 1 for correct responses and 0 for incorrect responses (Table 1). Non-response to knowledge questions was uncommon (<5%) due to interviewers’ diligence in avoiding skipped questions and encouraging hesitant respondents; when non-response did occur, data collection coordinators called or revisited providers to obtain missing data. Principal components analysis (PCA) was conducted on these variables in Stata 12.0 in order to construct three separate indexes: 1) a zinc knowledge index; 2) an ORS knowledge index; and 3) a combined zinc and ORS knowledge index [12].

**Table 1:** Binary variables of zinc and ORS knowledge.*

Survey question	Binary variables used to construct zinc knowledge index	Binary variables used to construct ORS knowledge index	Binary variables used to construct combined zinc AND ORS knowledge index
Reported treatment for a child with 5 loose/watery stools per day for 3 days and no dehydration	= 1 if Zinc; = 0 if would not treat child OR did not report zinc	= 1 if ORS; = 0 if would not treat child OR did not report ORS	= 1 if zinc AND ORS; = 0 if would not treat child OR did not report both zinc and ORS
Reported treatment for a child with 5 loose/watery stools per day for 3 days, sunken eyes and lethargy	= 1 if Zinc; = 0 if would not treat child OR did not report zinc	= 1 if ORS; = 0 if would not treat child OR did not report ORS	= 1 if zinc AND ORS; = 0 if would not treat child OR did not report both zinc and ORS
Reported treatment for a child with 4 loose/watery stools per day for 15 days	= 1 if Zinc; = 0 if would not treat child OR did not report zinc	= 1 if ORS; = 0 if would not treat child OR did not report ORS	= 1 if zinc AND ORS; = 0 if would not treat child OR did not report both zinc and ORS
Reported treatment for a child with bloody stools	= 1 if Zinc; = 0 if would not treat child OR did not report zinc	= 1 if ORS; = 0 if would not treat child OR did not report ORS	= 1 if zinc AND ORS; = 0 if would not treat child OR did not report both zinc and ORS
Reported zinc dose for child <6 months	= 1 if 10 mg for infants 2–5 months of age; = 0 if incorrect dose and/or age range specified**	-	= 1 if 10 mg for infants 2–5 months of age; = 0 if incorrect dose and/or age range specified**
Reported zinc dose for child 6–59 months	= 1 if 20 mg for children 6–59 months of age; = 0 if incorrect dose and/or age range specified**	-	= 1 if 20 mg for children 6–59 months of age; = 0 if incorrect dose and/or age range specified**
Reported zinc duration	= 1 if 10–14 days; = 0 if incorrect number of days	-	= 1 if 10–14 days; = 0 if incorrect number of days
Reported preparation of ORS for a child 2–59 months of age with diarrhea	-	= 1 if 1 L packet in 1 L water OR 200 mL packet in 1 cup water; = 0 if incorrect preparation	= 1 if 1 L packet in 1 L water OR 200 mL packet in 1 cup water; = 0 if incorrect preparation

*Contingency tables assessing the correlation between the binary variables used to construct the knowledge indexes are included in supplementary file S1 Appendix.

**Providers were not penalized for slight variations in the interpretation of age cut-offs. The following responses were also considered correct: (1) 10 mg for infants 2–6 months of age; (2) 20 mg for children 6–59 months/ 7–59 months/ 6–60 months/ 7–60 months of age

PCA is a method by which a set of correlated variables is reduced to a set of uncorrelated weighted linear combinations of the original variables [13]. PCA assigns a weight (also known as factor score) to each of the original variables, which is based upon the ability of that variable to explain variability across responses [13]. In other words, in constructing the knowledge indexes, higher PCA weights are allocated to variables derived from the survey questions that best differentiate providers with high and low knowledge. For example, if the majority of providers answer 6 out of 7 zinc knowledge questions correctly, variability in zinc knowledge is best assessed by responses to the 7^th^ question and that question will receive a higher PCA weight. Accordingly, variables with large splits in the frequency of correct and incorrect responses will be allocated higher PCA weights than more evenly distributed variables. The PCA weights thus provide an alternative to the more simplistic approach of uniformly assigning the same weight to all knowledge variables.

### Data analysis

Data analysis was designed to assess the relationship between the ORS/zinc/combined knowledge index scores and the outcomes of ORS prescribing, zinc prescribing, and combined ORS and zinc prescribing. Using data from the direct observation of each provider during a treatment interaction with a diarrhea case, we constructed binary outcome indicators of ORS/zinc prescribing. Prescribing was defined as having advised a specified treatment regardless of whether the product was directly provided during the consultation or advised through another channel; for example, in our analysis ASHAs and AWWs who did not have ORS/zinc in stock but advised a caregiver to obtain these products at the PHC were considered to have prescribed ORS/zinc.

For each sector, we used Stata 12.0 to fit three multiple logistic regression models of: 1) the log odds of observed zinc prescribing, 2) the log odds of observed ORS prescribing, and 3) the log odds of observed ORS and zinc prescribing [12]. All models included the relevant knowledge index score as the primary predictor variable and controlled for whether the provider had access to zinc and/or ORS supplies at the time of the observation. To identify additional covariates for inclusion, we employed a priori knowledge and conducted bivariate analyses, retaining variables with a p-value <0.10. The final models for both sectors included indicators of child age and sex, provider education, and receipt of diarrhea management training in the 6 months prior to the survey. Public sector models also included provider type (i.e. ASHA or AWW) and state (i.e. Bihar or Gujarat). We inspected the public sector models for interaction between state and access to ORS/zinc supplies using Wald tests of statistical significance; this interaction was considered because the ORS/zinc supply chain, including product procurement and distribution, varied by state. All regression analyses employed the robust cluster estimator of variance [14], where PHC was the cluster variable for public sector models and tehsil was the cluster variable for private sector models.

We employed the Hosmer and Lemeshow test to ensure the goodness-of-fit of each model by confirming p-values >0.05. We inspected final models for potential outliers and influential points by plotting leverage, studentized residuals and Cook’s distances. We subsequently performed sensitivity analyses, which confirmed that model coefficients were not statistically significantly altered by removal of extreme points and so they were retained.

## Results

### Provider characteristics and prescribing practices

Table 2 outlines the key demographic characteristics of included providers. As expected, all AWWs and ASHAs were female and all RMPs were male. On average, AWWs and ASHAs had 10 years of education and RMPs had 12 years of education. The majority of providers reported having attended diarrhea management training in the 6 months prior to the survey (i.e. 90% AWWs; 89% ASHAs; 69% RMPs).

**Table 2:** Reported and observed characteristics of AWWs, ASHAs and RMPs.

	AWW ^a^	ASHA ^a^	RMP ^a^
	(N = 330)	(N = 330)	(N = 97)
	n (%)	n (%)	n (%)
Sex			
Female	330 (100)	330 (100)	0
Male	0	0	97 (100)
Age (years): mean (SD)	36.7 (7.6)	31.4 (6.4)	41.1 (11.7)
Religion			
Hindu	308 (93.3)	315 (95.4)	82 (84.5)
Muslim	21 (6.4)	15 (4.6)	15 (15.5)
Education (years): median (max, min)	10 (4, 17)	10 (0, 17)	12 (9, 21)
Diarrhea training in the 6 months prior to survey			
Yes	297 (90.0)	295 (89.4)	67 (69.1)
Access to supplies at the time of survey			
ORS	145 (43.9)	191 (57.9)	70 (72.2)
Zinc	272 (82.4)	304 (92.1)	35 (36.1)
ORS and Zinc	129 (39.1)	182 (55.2)	33 (34.0)
Observed prescribing in consultation with diarrhea case ^b^			
ORS	244 (73.9)	278 (84.2)	55 (56.7)
Zinc	216 (65.5)	259 (78.5)	27 (28.1)
ORS and Zinc	216 (65.5)	257 (77.9)	19 (19.6)
Correct responses to zinc knowledge questions: ^c^			
Child with 5 loose/watery stools per day for 3 days and no dehydration	279 (84.5)	291 (88.2)	38 (39.2)
Child with 5 loose/watery stools per day for 3 days, sunken eyes and lethargy	95 (28.8)	109 (33.0)	30 (30.9)
Child with 4 loose/watery stools per day for 15 days and no dehydration	200 (60.6)	212 (64.2)	26 (26.8)
Child with bloody stools	113 (34.2)	140 (42.4)	23 (23.7)
10 mg zinc dose for child 2–5 months of age ^d^	115 (34.9)	115 (34.9)	6 (6.2)
20 mg zinc dose for child 6–59 months of age ^d^	155 (47.0)	165 (50.0)	6 (6.2)
10–14 days duration of zinc supplementation	278 (84.2)	292 (88.5)	52 (53.6)
Correct responses to ORS knowledge questions: ^e^			
Child with 5 loose/watery stools per day for 3 days and no dehydration	320 (97.0)	316 (95.8)	76 (78.4)
Child with 5 loose/watery stools per day for 3 days, sunken eyes and lethargy	110 (33.3)	120 (36.4)	53 (54.6)
Child with 4 loose/watery stools per day for 15 days and no dehydration	223 (67.6)	232 (70.3)	42 (43.3)
Child with bloody stools	123 (37.3)	158 (47.9)	44 (45.4)
ORS preparation: 1 L packet in 1 L water OR 200 mL packet in 1 cup water	309 (93.6)	319 (96.7)	50 (51.6)
Correct responses to zinc & ORS knowledge questions: ^f^			
Child with 5 loose/watery stools per day for 3 days and no dehydration	279 (84.6)	289 (87.6)	35 (36.1)
Child with 5 loose/watery stools per day for 3 days, sunken eyes and lethargy	95 (28.8)	109 (33.0)	28 (28.9)
Child with 4 loose/watery stools per day for 15 days and no dehydration	200 (60.6)	212 (64.2)	25 (25.8)
Child with bloody stools	113 (34.2)	138 (41.8)	15 (15.5)

^a^ AWWs and ASHAs were sampled from Bihar (N = 165 AWWs; N = 165 ASHAs) and Gujarat (N = 165 AWWs; N = 165 ASHAs); RMPs were sampled from UP.

^b^ Prescribing was defined as having advised a specified treatment regardless of whether the product was directly provided during the consultation or advised through another channel.

^c^ N (%) responding zinc

^d^ Providers not penalized for slight variations in the interpretation of age cut-offs. The following responses were also considered correct: (1) 10 mg for infants 2–6 months of age; (2) 20 mg for children 6–59 months/ 7–59 months/ 6–60 months/ 7–60 months of age

^e^ N (%) responding ORS

^f^ N (%) responding zinc AND ORS

At the time of the survey, both ORS and zinc supplies were accessible to 39%, 55% and 34% of AWWs, ASHAs and RMPs, respectively. During direct observation, both zinc and ORS were advised by more than 65% of AWWs and nearly 78% of ASHAs but only 19.6% of RMPs. ORS was prescribed without zinc by 28 (8.5%) AWWs, 21 (6.4%) ASHAs, and 36 (37.1%) RMPs. Zinc was prescribed without ORS by 2 (0.6%) ASHAs and 8 (8.2%) RMPs.

### Knowledge Index Scores

Table 2 lists the proportion of providers that responded correctly to each question included in construction of the index scores, and Table 3 summarizes the PCA-derived weights (*f*_*n*_) that were applied to each variable during construction of knowledge indexes for the public and private sectors. The positive value of all weights indicates that correct responses to all knowledge questions were associated with higher knowledge index scores. Higher weights were applied to questions with greater variability across responses (i.e. questions that differentiated between providers with high and low levels of ORS, zinc or combined ORS and zinc knowledge). For example, knowledge of the correct duration of zinc treatment received the highest weight in construction of the zinc knowledge index score for the public sector (weight = 0.71); therefore, knowledge of zinc duration can be used to differentiate high and low achievers.

**Table 3:** Results of principal components analysis (PCA) for public and private sector models.*

Binary variable	Weights used in construction of zinc knowledge index score	Weights used in construction of ORS knowledge index score	Weights used in construction of combined zinc AND ORS knowledge index score
**Public Sector**			
Reported treatment for a child with 5 loose/watery stools per day for 3 days and no dehydration	0.67	0.38	0.67
Reported treatment for a child with 5 loose/watery stools per day for 3 days, sunken eyes and lethargy	0.36	0.56	0.35
Reported treatment for a child with 4 loose/watery stools per day for 15 days	0.55	0.71	0.53
Reported treatment for a child with bloody stools	0.40	0.70	0.39
Reported zinc dose for child 2–5 months	0.59	-	0.57
Reported zinc dose for child 6–59 months	0.59	-	0.57
Reported zinc duration	0.71	-	0.71
Reported preparation of ORS for a child 2–59 months of age with diarrhea	-	0.21	0.38
**Private Sector**			
Reported treatment for a child with 5 loose/watery stools per day for 3 days and no dehydration	0.69	0.46	0.63
Reported treatment for a child with 5 loose/watery stools per day for 3 days, sunken eyes and lethargy	0.78	0.76	0.74
Reported treatment for a child with 4 loose/watery stools per day for 15 days	0.68	0.73	0.70
Reported treatment for a child with bloody stools	0.77	0.59	0.72
Reported zinc dose for child 2–5 months	0.11	-	0.24
Reported zinc dose for child 6–59 months	0.07	-	0.21
Reported zinc duration	0.37	-	0.35
Reported preparation of ORS for a child 2–59 months of age with diarrhea	-	0.12	0.14

*The PCA-generated weights are the eigenvectors of the first principal component; eigenvectors were derived from the correlation matrix in Stata 12.0 [12, 13]. The first principal component explained 32% and 33% of the variance in the data for the public and private sector knowledge indexes, respectively.

**Fig 1:**
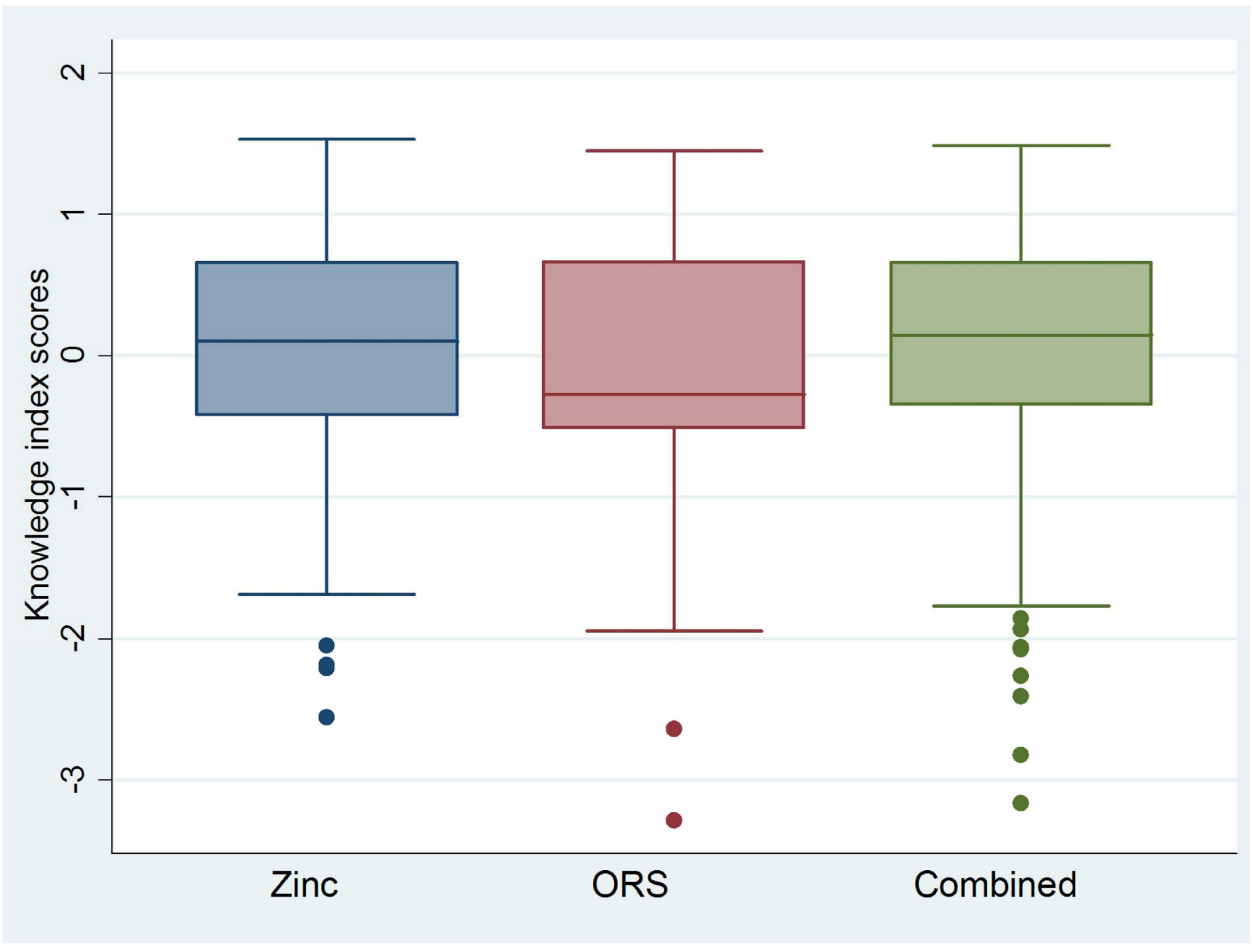
Boxplots* showing ORS, zinc and combined ORS and zinc knowledge index scores for public sector ASHAs and AWWs. *Boxplots are centered on the mean, which is approximately zero, and display a horizontal line at the median. ** Skewness and kurtosis estimates generated in Stata 12.0 [12]: Zinc (-0.91; 3.56); ORS (-0.40; 2.81); Combined (-1.08; 4.14).

**Fig 2:**
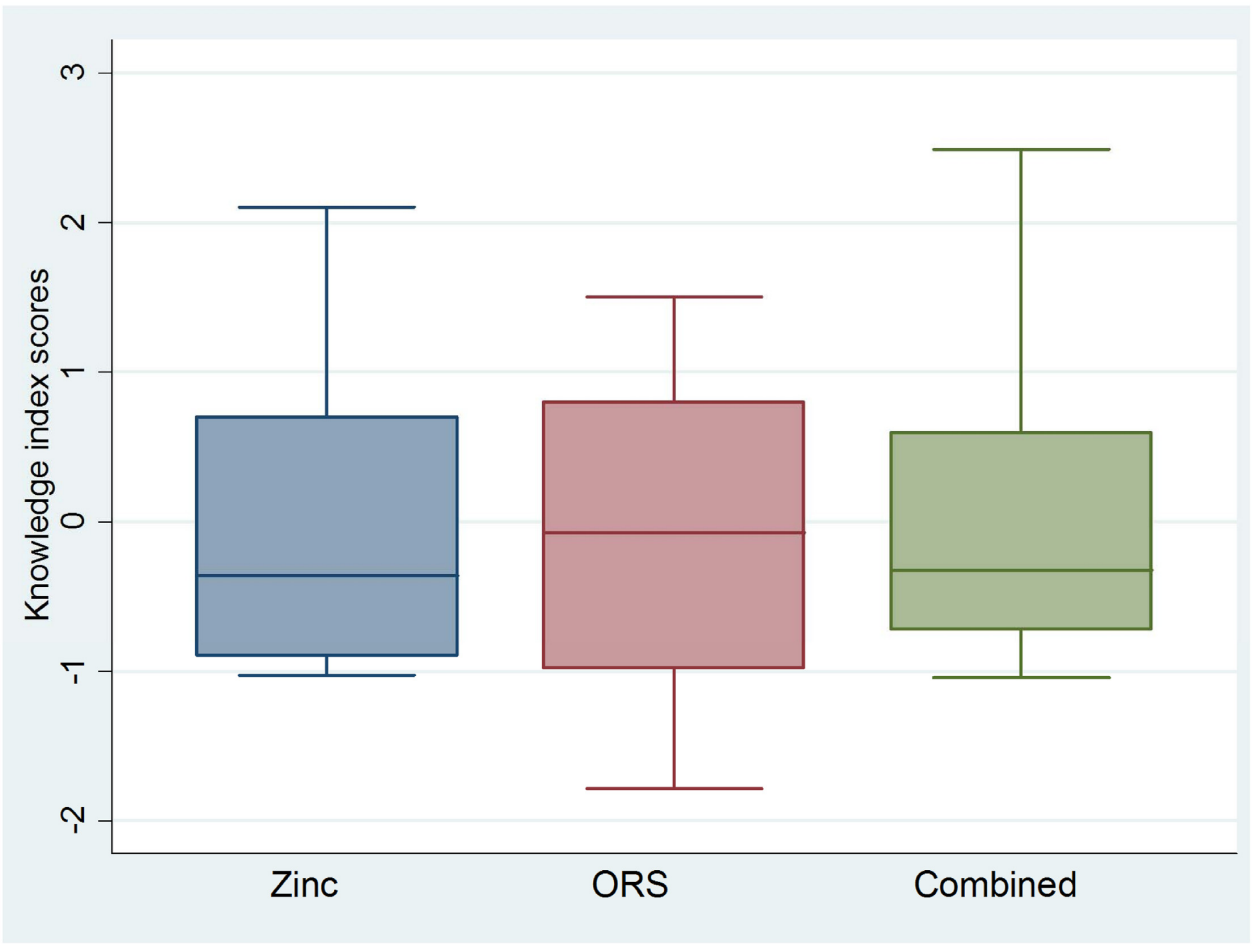
Boxplots* showing ORS, zinc and combined ORS and zinc knowledge index scores for private sector RMPs. *Boxplots are centered on the mean, which is approximately zero, and display a horizontal line at the median. ** Skewness and kurtosis estimates generated in Stata 12.0 [12]: Zinc (0.72; 2.29); ORS (-0.05; 1.93); Combined (0.92; 2.89).

In general, the distributions of knowledge index scores were approximately normal, an expected result of PCA [15], but showed slight negative skew among ASHAs/AWWs and slight positive skew among RMPs (Figs 1 and 2). These skew patterns indicate that providers achieving low knowledge scores were outliers among public sector ASHAs/AWWs; whereas, RMPs with high scores deviated from the expected level of knowledge of their RMP counterparts.

### Public Sector Regression Analyses

In the public sector analyses (Table 4), the adjusted odds of zinc prescribing were about two-fold higher per point increase in the zinc knowledge index score (aOR: 2.14; 95% CI: 1.70–2.70); the adjusted odds of ORS prescribing were 54% higher per unit increase in the ORS knowledge index score (aOR: 1.54; 95% CI: 1.24–1.92); and the adjusted odds of prescribing both ORS and zinc were elevated by a factor of 2.48 per unit increase in the combined ORS and zinc knowledge index score (aOR: 2.48; 95% CI: 1.90–3.24). In all three models, the adjusted odds of prescribing were higher among ASHAs compared to AWWs, providers with access to ORS/zinc at the time of the survey, and providers who had received diarrhea management training in the 6 months prior to the survey. There was a non-statistically significant trend towards increased adjusted odds of prescribing among ASHAs/AWWs with >10 years of schooling. The adjusted odds of prescribing zinc were reduced by 48% (95% CI: 11–69%) for children <6 months compared to those 6–59 months of age, and a similar trend was observed for ORS prescribing (aOR: 0.66; 95% CI: 0.40–1.10). Compared to Gujarat, the adjusted odds of zinc prescribing were elevated by a factor of 3.14 (95% CI: 1.56–6.34) in Bihar, but state was not a statistically significant predictor of ORS prescribing. The models did not show evidence of an interaction between state and access to ORS/zinc supplies.

**Table 4:** Factors associated with prescribing among public sector ASHAs and AWWs.

Variable	Adjusted OR ^a^	95% CI	P-value
**Outcome: Prescribing both ORS and zinc**			
ORS and zinc combined knowledge index score	2.48	1.90–3.24	<<0.001
Provider type			
ASHA	1.61	1.05–2.48	0.029
AWW	1.0		
Access to both ORS and zinc at the time of the survey	4.82	1.99–11.71	0.001
Received diarrhea management training in the 6 months prior to the survey	3.96	2.03–7.72	<<0.001
Provider education ^b^			
>10 years of school	1.30	0.82–2.08	0.264
≤ 10 years of school	1.0		
Observed child age			
< 6 months	0.61	0.37–1.01	0.055
6–59 months	1.0		
Observed child sex			
Male	1.09	0.74–1.58	0.670
Female	1.0		
State			
Bihar	0.56	0.23–1.37	0.202
Gujarat	1.0		
**Outcome: Prescribing ORS**			
ORS knowledge index score	1.54	1.24–1.92	<<0.001
Provider type			
ASHA	1.64	1.02–2.66	0.042
AWW	1.0		
Access to ORS at the time of the survey	6.96	2.75–17.61	<<0.001
Received diarrhea management training in the 6 months prior to the survey	4.00	2.16–7.38	<<0.001
Provider education ^b^			
>10 years of school	1.15	0.70–1.91	0.578
≤ 10 years of school	1.0		
Observed child age			
< 6 months	0.66	0.40–1.10	0.109
6–59 months	1.0		
Observed child sex			
Male	1.18	0.78–1.78	0.445
Female	1.0		
State			
Bihar	0.58	0.24–1.40	0.225
Gujarat	1.0		
**Outcome: Prescribing zinc**			
Zinc knowledge index score	2.14	1.70–2.70	<<0.001
Provider type			
ASHA	1.74	1.10–2.75	0.018
AWW	1.0		
Access to zinc at the time of the survey	17.42	6.59–46.06	<<0.001
Received diarrhea management training in the 6 months prior to the survey	3.07	1.64–5.76	<<0.001
Provider education ^b^			
>10 years of school	1.26	0.79–2.00	0.336
≤ 10 years of school	1.0		
Observed child age			
< 6 months	0.52	0.31–0.89	0.016
6–59 months	1.0		
Observed child sex			
Male	1.09	0.74–1.61	0.665
Female	1.0		
State			
Bihar	3.14	1.56–6.34	0.001
Gujarat	1.0		

^a^ Estimates were calculated using logistic regression with the robust cluster estimator of variance in Stata 12.0 [12].

^b^ Threshold of dichotomous variable for education was set at the mean number of years of schooling among ASHAs and AWWs (i.e. 10 years).

### Private Sector Regression Analyses

In the private sector analyses (Table 5), the adjusted odds of zinc prescribing increased by a factor of 3.57 (95% CI: 2.08–6.13) per unit increase in the zinc knowledge index score. The adjusted odds of ORS prescribing were not significantly associated with the ORS knowledge index score (aOR: 1.28; 95% CI: 0.79–2.07); however, the adjusted odds of prescribing both ORS and zinc increased by a factor of 2.32 (95% CI: 1.29–4.17) per one-point increase in the combined ORS and zinc knowledge index score.

**Table 5:** Factors associated with prescribing among private sector RMPs.

Variable	Adjusted OR ^a^	95% CI	P-value
**Outcome: Prescribing both ORS and zinc**			
ORS and zinc combined knowledge index score	2.32	1.29–4.17	0.005
Access to both ORS and zinc at the time of the survey	1.42	0.37–5.51	0.613
Received diarrhea management training in the 6 months prior to the survey	0.88	0.24–3.25	0.844
Provider education ^b^			
>14 years of school	3.31	0.96–11.38	0.057
≤ 14 years of school	1.0		
Observed child age			
< 6 months	3.82	0.39–37.18	0.248
6–59 months	1.0		
Observed child sex			
Male	1.90	0.40–8.97	0.420
Female	1.0		
**Outcome: Prescribing ORS**			
ORS knowledge index score	1.28	0.79–2.07	0.318
Access to ORS at the time of the survey	8.56	2.81–26.11	<<0.001
Received diarrhea management training in the 6 months prior to the survey	0.96	0.33–2.76	0.942
Provider education ^b^			
>14 years of school	0.62	0.21–1.82	0.384
≤ 14 years of school	1.0		
Observed child age			
< 6 months	2.06	0.21–20.33	0.537
6–59 months	1.0		
Observed child sex			
Male	1.37	0.51–3.67	0.528
Female	1.0		
**Outcome: Prescribing zinc**			
Zinc knowledge index score	3.57	2.08–6.13	<<0.001
Access to zinc at the time of the survey	0.60	0.16–2.26	0.452
Received diarrhea management training in the 6 months prior to the survey	1.61	0.45–5.71	0.464
Provider education ^b^			
>14 years of school	4.24	1.34–13.40	0.014
≤ 14 years of school	1.0		
Observed child age			
< 6 months	2.02	0.30–13.47	0.466
6–59 months	1.0		
Observed child sex			
Male	1.44	0.36–5.71	0.606
Female	1.0		

^a^ Estimates were calculated using logistic regression with the robust cluster estimator of variance in Stata 12.0 [12].

^b^ Threshold of dichotomous variable for education was set at the mean number of years of schooling among RMPs (i.e. 14 years).

Access to ORS supplies at the time of the survey was associated with higher adjusted odds of prescribing ORS (aOR: 8.56; 95% CI: 2.81–26.11), but access to zinc supplies did not have an effect on zinc prescribing (aOR: 0.60; 95% CI: 0.16–2.26). Receipt of diarrhea management training in the 6 months prior to the survey did not have a statistically significant effect on prescribing zinc (aOR: 1.61; 95% CI: 0.45–5.71), ORS (aOR: 0.96; 95% CI: 0.33–2.76) or both zinc and ORS (aOR: 0.88; 95% CI: 0.24–3.25). The adjusted odds of prescribing zinc were 4.24 times higher (95% CI: 1.34–13.40) among RMPs with >14 years of education; however, education did not have an effect on ORS prescribing (aOR: 0.62; 95% CI: 0.21–1.82). In all three models, there was a non-statistically significant trend towards higher odds of prescribing ORS/zinc for children <6 months compared to 6–59 months of age.

## Discussion

The practice of advising zinc and ORS for diarrhea treatment among children under-five was positively influenced by zinc and ORS knowledge as measured by a novel scale. In the construction of the knowledge score indexes, the allocation of PCA weights to survey items allowed for greater weighting of the questions that best differentiated providers with high and low levels of zinc and ORS knowledge and was thus advantageous over simply assigning uniform weight to all knowledge questions. Furthermore, the use of PCA weighting led to the determination that the weighting of knowledge questions should differ between public and private sector providers, as the most heavily weighted items differed between the public and private sector indexes (Table 3): accurate knowledge of zinc dose and duration and of the need to advise ORS for persistent and bloody diarrhea received the greatest weights in the construction of the public sector indexes; whereas, in construction of the private sector indexes, the greatest weights were awarded to accurate knowledge of the need to advise zinc and ORS for mild and severe diarrheal episodes.

Receipt of training in the 6 months prior to the survey was associated with higher odds of ORS and zinc prescribing among ASHAs and AWWs regardless of knowledge score (Table 4), suggesting the importance of periodic reinforcement of diarrhea management curriculum even among providers with high knowledge of ORS and zinc. As such, diarrhea management programs should ensure supervision or refresher training on at least a biannual basis for all ASHAs and AWWs.

Training of private sector RMPs, on the other hand, had no effect on observed prescribing practices after controlling for knowledge and other factors (Table 5). Unlike public sector providers, RMPs were not formally trained but instead received visits from pharmaceutical or NGO representatives promoting adequate treatment of childhood diarrhea while simultaneously soliciting sales of zinc and ORS. It is probable that most representatives interacted with RMPs for a short period of time; whereas, diarrhea management training of ASHAs and AWWs was comprised of formal class sessions lasting hours. It is therefore conceivable that the nature of RMP training precluded a measurable association with prescribing practices. In addition, the small number of RMPs that reported receiving training in the 6 months prior to the survey may have also prevented an association. The small sample size of trained RMPs may be indicative of the hurdles pharmaceutical and NGO representatives encountered when attempting to reach RMPs, since these informal practitioners often operate underground in an attempt to evade government regulations. It is also possible that RMPs had poor recall of visits to their practices, which were of short duration and may not have been especially memorable. These findings suggest that the current structure of RMP training is not effective. Still, it is improbable that communal diarrhea management training sessions, which have had demonstrated impact on prescribing practices among public sector ASHAs and AWWs, would be logistically feasible for RMPs. Further research is warranted to assess whether changing the structure or increasing the duration and/or frequency of one-on-one RMP training sessions can improve RMP prescribing practices for diarrhea among children under-five.

The odds of prescribing ORS and zinc were highly elevated among ASHAs and AWWs with direct access to ORS and zinc supplies, controlling for knowledge score and other factors (Table 4). In the event of stock-outs, ASHAs and AWWs are trained to advise ORS and zinc anyway and to refer caregivers to the PHC to obtain these products. Our findings, however, demonstrate that in the absence of ORS and zinc stocks, ASHAs and AWWs were less likely to mention the products during the treatment consultation. Stock-outs are thus a significant impediment to adequate diarrhea treatment in the community. To help overcome this challenge, trainings of ASHAs and AWWs should emphasize the importance of referring caregivers to obtain ORS and zinc through other channels if supplies are not directly available. Still, a system of referrals is not a substitute for a reliable supply chain, which is a necessity in order to ensure sustainable access to ORS and zinc in the public sector in India.

Among RMPs, there was a strong association between direct access to ORS supplies at the time of the survey and ORS prescribing (Table 5). This finding is important because prior to program implementation, RMPs generally did not stock or advise ORS, and the relationship between access and prescribing suggests that increasing the availability of ORS products at RMP practices can help alter RMP prescribing behavior. Still, RMPs commonly advise medications through other channels, such as affiliated local chemists and drug stores, which may explain why direct access to zinc at the time of the survey had no effect on zinc prescribing after controlling for other factors (Table 5). Diarrhea management programs should therefore expand the focus of private sector implementation to include chemists and drug stores in an effort to promote broad access to ORS and zinc across the private sector.

ASHAs prescribed ORS and zinc with higher odds than AWWs. Although total years of education and the training curricula for ASHAs and AWWs were identical, it is possible that ASHAs were more comfortable in the role of distributing treatment, since they have traditionally carried medical kits and AWWs have only recently begun to fulfill this expectation. Diarrhea management programs operating in the public sector should attempt to build AWWs’ capacity and confidence for advising and dispensing treatment.

The internal validity of this study may be limited by the Hawthorne effect, which results when the presence of an outside observer causes an individual to deviate from his or her normal behavior [16]. It is thus possible that the presence of study staff during the observed diarrhea consultation led providers to alter their treatment and prescribing practices. However, due to the overall low frequency of ORS and zinc prescribing compared to reported knowledge during interview, it is unlikely that providers were aware of the observers’ research agenda; while providers may have modified their behavior due to the presence of study staff, it is unlikely they adjusted their practices concerning ORS and zinc prescribing—the main study outcomes, thereby reducing the threat to internal validity.

The external validity of this study is dependent upon health system structure and geography, and as such, our findings may be generalizable to other low resource settings that require simultaneous private and public sector scale-up of ORS and zinc. Conclusions regarding the public sector are relevant in regions where community health workers similar to ASHAs and AWWs are accountable for a large portion of the diarrhea treatment provided under the government health system. Findings concerning RMPs are applicable to places in which a substantial amount of careseeking and treatment for childhood diarrhea occurs through a network of informal providers who are engrained in the community but whose roles have not necessarily been recognized by the overarching, formal health system. Implementers of future efforts to scale-up ORS and zinc in similar contexts, especially in other Indian states, can therefore utilize our findings to glean appropriate lessons on the importance of provider trainings and the role of specific factors in increasing ORS and zinc prescribing practices.

## Supporting Information

S1 Appendix Assessment of the correlation between binary variables used to construct knowledge indexes. (DOCX)

## References

[1] Liu L, Oza S, Hogan D, Perin J, Rudan I, Lawn JE, et al Global, regional, and national causes of child mortality in 2000–13, with projections to inform post-2015 priorities: an updated systematic analysis. The Lancet. 2014;[Epub ahead of print]. 10.1016/S0140-6736(14)61698-6 25280870

[2] Liu L, Johnson HL, Cousens S, Perin J, Scott S, Lawn JE, et al Global, regional, and national causes of child mortality: an updated systematic analysis for 2010 with time trends since 2000. Lancet. 2012;379(9832):2151–61. Epub 2012/05/15. 10.1016/S0140-6736(12)60560-1 . 22579125

[3] Bhatnagar S, Lodha R, Choudhury P, Sachdev HP, Shah N, Narayan S, et al IAP Guidelines 2006 on management of acute diarrhea. Indian Pediatr. 2007;44:380–9. 17536143

[4] IAP. IAP Guidelines on management of diarrhoea 2008. Available: http://www.pediatriconcall.com/fordoctor/diarrhea/iap_guidelines.asp.

[5] Nath I, Reddy KS, Dinshaw KA, Bhisey AN, Krishnaswami K, Bhan MK, et al Country profile: India. Lancet. 1998;351(9111):1265–75. Epub 1998/06/27. doi: S0140673698030104 [pii]. . 9643763 10.1016/s0140-6736(98)03010-4

[6] IIPS (International Institute for Population Sciences) and Macro International. National Family Health Survey (NFHS-3), 2005–06, India: Key Findings. Mumbai: IIPS, 2007.

[7] Kumar R, Jaiswal V, Tripathi S, Kumar A, Idris MZ. Inequity in health care delivery in India: the problem of rural medical practitioners. Health Care Anal. 2007;15(3):223–33. Epub 2007/10/09. 10.1007/s10728-007-0060-x . 17922199

[8] George A, Iyer A, Sen G. Unregulated but Unfree Market Relations: Ambivalent Views from Informal Providers in Rural South India. [Presentation]. In press 2011.

[9] Kanjilal B, Mondal S, Samanta T, Mondal A, Singh S. A Parallel Health Care Market: Rural Medical Practitioners in West Bengal, India. Jaipur, India: Institute of Health Management Research, 2007.

[10] Sillah F, Ho HJ, Chao JC. The use of oral rehydration salt in managing children under 5 y old with diarrhea in the Gambia: Knowledge, attitude, and practice. Nutrition. 2013;29(11–12):1368–73. Epub 2013/10/10. 10.1016/j.nut.2013.05.014 . 24103515

[11] Pham DM, Byrkit M, Pham HV, Pham T, Nguyen CT. Improving Pharmacy Staff Knowledge and Practice on Childhood Diarrhea Management in Vietnam: Are Educational Interventions Effective? PLoS One. 2013;8(10):e74882 Epub 2013/10/08. 10.1371/journal.pone.0074882 24098355 PMC3789740

[12] StataCorp. 2011. Stata Statistical Software: Release 12. College Station TX: StataCorp LP.

[13] Vyas S, Kumaranayake L. Constructing socio-economic status indices: how to use principal components analysis. Health Policy Plan. 2006;21(6):459–68. Epub 2006/10/13. czl029 [pii] 10.1093/heapol/czl029 . 17030551

[14] Williams RL. A note on robust variance estimation for cluster-correlated data. Biometrics. 2000;56(2):645–6. . 10877330 10.1111/j.0006-341x.2000.00645.x

[15] Kolenikov K, Angeles G. The use of discrete data in PCA: Theory, simulations, and applications to socioeconomic indices. Chapel Hill, NC: University of North Carolina 2005.

[16] Mayo E. The human problems of an industrial civilization. New York: MacMillan; 1933.

