# Supplementary material for: The Association between Provider Practice and Knowledge of ORS and Zinc Supplementation for the Treatment of Childhood Diarrhea in Bihar, Gujarat and Uttar Pradesh, India: A Multi-Site Cross-Sectional Study
Source: PLoS One. 2015 Jun 22;10(6):e0130845. doi: 10.1371/journal.pone.0130845 (PMC4476718; doi:10.1371/journal.pone.0130845)
Supplement: S1 Appendix — (DOCX) [file pone.0130845.s001.docx]

**S1 Appendix. Assessment of the correlation between binary variables used to construct knowledge indexes**

S1 Appendix A. Key showing variable names used in the following analyses

| **Survey question** | **Binary variables used to construct zinc knowledge index** | **Binary variables used to construct ORS knowledge index** | **Binary variables used to construct combined zinc AND ORS knowledge index** |
| --- | --- | --- | --- |
| Reported treatment for a child with 5 loose/watery stools per day for 3 days and no dehydration | ZINC1 | ORS1 | ZINCORS1 |
| Reported treatment for a child with 5 loose/watery stools per day for 3 days, sunken eyes and lethargy | ZINC2 | ORS2 | ZINCORS2 |
| Reported treatment for a child with 4 loose/watery stools per day for 15 days | ZINC3 | ORS3 | ZINCORS3 |
| Reported treatment for a child with bloody stools | ZINC4 | ORS4 | ZINCORS4 |
| Reported zinc dose for child <6 months | ZINCDOSE_6MOS | - | ZINCDOSE_6MOS |
| Reported zinc dose for child 6-59 months | ZINCDOSE_59MOS | - | ZINCDOSE_59MOS |
| Reported zinc duration | ZINCDURATION | - | ZINCDURATION |
| Reported preparation of ORS for a child 2-59 months of age with diarrhea | - | ORSPREP | ORSPREP |

S1 Appendix B. Contingency Tables – Public Sector

| ORS2

ORS1 | 0 1 | Total

-----------+----------------------+----------

0 | 19 5 | 24

| 4.42 2.17 | 3.64

-----------+----------------------+----------

1 | 411 225 | 636

| 95.58 97.83 | 96.36

-----------+----------------------+----------

Total | 430 230 | 660

| 100.00 100.00 | 100.00

Pearson chi2(1) = 2.1547 Pr = 0.142

| ORS3

ORS1 | 0 1 | Total

-----------+----------------------+----------

0 | 14 10 | 24

| 6.83 2.20 | 3.64

-----------+----------------------+----------

1 | 191 445 | 636

| 93.17 97.80 | 96.36

-----------+----------------------+----------

Total | 205 455 | 660

| 100.00 100.00 | 100.00

Pearson chi2(1) = 8.6512 Pr = 0.003

| ORS4

ORS1 | 0 1 | Total

-----------+----------------------+----------

0 | 18 6 | 24

| 4.75 2.14 | 3.64

-----------+----------------------+----------

1 | 361 275 | 636

| 95.25 97.86 | 96.36

-----------+----------------------+----------

Total | 379 281 | 660

| 100.00 100.00 | 100.00

Pearson chi2(1) = 3.1468 Pr = 0.076

| ORSPREP

ORS1 | 0 1 | Total

-----------+----------------------+----------

0 | 5 19 | 24

| 15.63 3.03 | 3.64

-----------+----------------------+----------

1 | 27 609 | 636

| 84.38 96.97 | 96.36

-----------+----------------------+----------

Total | 32 628 | 660

| 100.00 100.00 | 100.00

Pearson chi2(1) = 13.7941 Pr = 0.000

| ORS3

ORS2 | 0 1 | Total

-----------+----------------------+----------

0 | 159 271 | 430

| 77.56 59.56 | 65.15

-----------+----------------------+----------

1 | 46 184 | 230

| 22.44 40.44 | 34.85

-----------+----------------------+----------

Total | 205 455 | 660

| 100.00 100.00 | 100.00

Pearson chi2(1) = 20.1690 Pr = 0.000

| ORS4

ORS2 | 0 1 | Total

-----------+----------------------+----------

0 | 275 155 | 430

| 72.56 55.16 | 65.15

-----------+----------------------+----------

1 | 104 126 | 230

| 27.44 44.84 | 34.85

-----------+----------------------+----------

Total | 379 281 | 660

| 100.00 100.00 | 100.00

Pearson chi2(1) = 21.5156 Pr = 0.000

| ORSPREP

ORS2 | 0 1 | Total

-----------+----------------------+----------

0 | 25 405 | 430

| 78.13 64.49 | 65.15

-----------+----------------------+----------

1 | 7 223 | 230

| 21.88 35.51 | 34.85

-----------+----------------------+----------

Total | 32 628 | 660

| 100.00 100.00 | 100.00

Pearson chi2(1) = 2.4931 Pr = 0.114

| ORS4

ORS3 | 0 1 | Total

-----------+----------------------+----------

0 | 165 40 | 205

| 43.54 14.23 | 31.06

-----------+----------------------+----------

1 | 214 241 | 455

| 56.46 85.77 | 68.94

-----------+----------------------+----------

Total | 379 281 | 660

| 100.00 100.00 | 100.00

Pearson chi2(1) = 64.6966 Pr = 0.000

| ORSPREP

ORS3 | 0 1 | Total

-----------+----------------------+----------

0 | 10 195 | 205

| 31.25 31.05 | 31.06

-----------+----------------------+----------

1 | 22 433 | 455

| 68.75 68.95 | 68.94

-----------+----------------------+----------

Total | 32 628 | 660

| 100.00 100.00 | 100.00

Pearson chi2(1) = 0.0006 Pr = 0.981

| ORSPREP

ORS4 | 0 1 | Total

-----------+----------------------+----------

0 | 20 359 | 379

| 62.50 57.17 | 57.42

-----------+----------------------+----------

1 | 12 269 | 281

| 37.50 42.83 | 42.58

-----------+----------------------+----------

Total | 32 628 | 660

| 100.00 100.00 | 100.00

Pearson chi2(1) = 0.3544 Pr = 0.552

| ZINC2

ZINC1 | 0 1 | Total

-----------+----------------------+----------

0 | 81 9 | 90

| 17.76 4.41 | 13.64

-----------+----------------------+----------

1 | 375 195 | 570

| 82.24 95.59 | 86.36

-----------+----------------------+----------

Total | 456 204 | 660

| 100.00 100.00 | 100.00

Pearson chi2(1) = 21.3341 Pr = 0.000

| ZINC3

ZINC1 | 0 1 | Total

-----------+----------------------+----------

0 | 69 21 | 90

| 27.82 5.10 | 13.64

-----------+----------------------+----------

1 | 179 391 | 570

| 72.18 94.90 | 86.36

-----------+----------------------+----------

Total | 248 412 | 660

| 100.00 100.00 | 100.00

Pearson chi2(1) = 67.8894 Pr = 0.000

| ZINC4

ZINC1 | 0 1 | Total

-----------+----------------------+----------

0 | 76 14 | 90

| 18.67 5.53 | 13.64

-----------+----------------------+----------

1 | 331 239 | 570

| 81.33 94.47 | 86.36

-----------+----------------------+----------

Total | 407 253 | 660

| 100.00 100.00 | 100.00

Pearson chi2(1) = 22.8722 Pr = 0.000

| ZINCDOSE_6MOS

ZINC1 | 0 1 | Total

-----------+----------------------+----------

0 | 75 15 | 90

| 17.44 6.52 | 13.64

-----------+----------------------+----------

1 | 355 215 | 570

| 82.56 93.48 | 86.36

-----------+----------------------+----------

Total | 430 230 | 660

| 100.00 100.00 | 100.00

Pearson chi2(1) = 15.1732 Pr = 0.000

| ZINCDOSE_59MOS

ZINC1 | 0 1 | Total

-----------+----------------------+----------

0 | 66 24 | 90

| 19.41 7.50 | 13.64

-----------+----------------------+----------

1 | 274 296 | 570

| 80.59 92.50 | 86.36

-----------+----------------------+----------

Total | 340 320 | 660

| 100.00 100.00 | 100.00

Pearson chi2(1) = 19.8613 Pr = 0.000

| ZINCDURATION

ZINC1 | 0 1 | Total

-----------+----------------------+----------

0 | 48 42 | 90

| 53.33 7.37 | 13.64

-----------+----------------------+----------

1 | 42 528 | 570

| 46.67 92.63 | 86.36

-----------+----------------------+----------

Total | 90 570 | 660

| 100.00 100.00 | 100.00

Pearson chi2(1) = 139.4430 Pr = 0.000

| ZINC3

ZINC2 | 0 1 | Total

-----------+----------------------+----------

0 | 205 251 | 456

| 82.66 60.92 | 69.09

-----------+----------------------+----------

1 | 43 161 | 204

| 17.34 39.08 | 30.91

-----------+----------------------+----------

Total | 248 412 | 660

| 100.00 100.00 | 100.00

Pearson chi2(1) = 34.2591 Pr = 0.000

| ZINC4

ZINC2 | 0 1 | Total

-----------+----------------------+----------

0 | 313 143 | 456

| 76.90 56.52 | 69.09

-----------+----------------------+----------

1 | 94 110 | 204

| 23.10 43.48 | 30.91

-----------+----------------------+----------

Total | 407 253 | 660

| 100.00 100.00 | 100.00

Pearson chi2(1) = 30.3512 Pr = 0.000

| ZINCDOSE_6MOS

ZINC2 | 0 1 | Total

-----------+----------------------+----------

0 | 302 154 | 456

| 70.23 66.96 | 69.09

-----------+----------------------+----------

1 | 128 76 | 204

| 29.77 33.04 | 30.91

-----------+----------------------+----------

Total | 430 230 | 660

| 100.00 100.00 | 100.00

Pearson chi2(1) = 0.7531 Pr = 0.386

| ZINCDOSE_59MOS

ZINC2 | 0 1 | Total

-----------+----------------------+----------

0 | 229 227 | 456

| 67.35 70.94 | 69.09

-----------+----------------------+----------

1 | 111 93 | 204

| 32.65 29.06 | 30.91

-----------+----------------------+----------

Total | 340 320 | 660

| 100.00 100.00 | 100.00

Pearson chi2(1) = 0.9919 Pr = 0.319

| ZINCDURATION

ZINC2 | 0 1 | Total

-----------+----------------------+----------

0 | 79 377 | 456

| 87.78 66.14 | 69.09

-----------+----------------------+----------

1 | 11 193 | 204

| 12.22 33.86 | 30.91

-----------+----------------------+----------

Total | 90 570 | 660

| 100.00 100.00 | 100.00

Pearson chi2(1) = 17.0403 Pr = 0.000

| ZINC4

ZINC3 | 0 1 | Total

-----------+----------------------+----------

0 | 209 39 | 248

| 51.35 15.42 | 37.58

-----------+----------------------+----------

1 | 198 214 | 412

| 48.65 84.58 | 62.42

-----------+----------------------+----------

Total | 407 253 | 660

| 100.00 100.00 | 100.00

Pearson chi2(1) = 85.8969 Pr = 0.000

| ZINCDOSE_6MOS

ZINC3 | 0 1 | Total

-----------+----------------------+----------

0 | 178 70 | 248

| 41.40 30.43 | 37.58

-----------+----------------------+----------

1 | 252 160 | 412

| 58.60 69.57 | 62.42

-----------+----------------------+----------

Total | 430 230 | 660

| 100.00 100.00 | 100.00

Pearson chi2(1) = 7.6746 Pr = 0.006

| ZINCDOSE_59MOS

ZINC3 | 0 1 | Total

-----------+----------------------+----------

0 | 135 113 | 248

| 39.71 35.31 | 37.58

-----------+----------------------+----------

1 | 205 207 | 412

| 60.29 64.69 | 62.42

-----------+----------------------+----------

Total | 340 320 | 660

| 100.00 100.00 | 100.00

Pearson chi2(1) = 1.3565 Pr = 0.244

| ZINCDURATION

ZINC3 | 0 1 | Total

-----------+----------------------+----------

0 | 57 191 | 248

| 63.33 33.51 | 37.58

-----------+----------------------+----------

1 | 33 379 | 412

| 36.67 66.49 | 62.42

-----------+----------------------+----------

Total | 90 570 | 660

| 100.00 100.00 | 100.00

Pearson chi2(1) = 29.4755 Pr = 0.000

| ZINCDOSE_6MOS

ZINC4 | 0 1 | Total

-----------+----------------------+----------

0 | 266 141 | 407

| 61.86 61.30 | 61.67

-----------+----------------------+----------

1 | 164 89 | 253

| 38.14 38.70 | 38.33

-----------+----------------------+----------

Total | 430 230 | 660

| 100.00 100.00 | 100.00

Pearson chi2(1) = 0.0196 Pr = 0.889

| ZINCDOSE_59MOS

ZINC4 | 0 1 | Total

-----------+----------------------+----------

0 | 212 195 | 407

| 62.35 60.94 | 61.67

-----------+----------------------+----------

1 | 128 125 | 253

| 37.65 39.06 | 38.33

-----------+----------------------+----------

Total | 340 320 | 660

| 100.00 100.00 | 100.00

Pearson chi2(1) = 0.1397 Pr = 0.709

| ZINCDURATION

ZINC4 | 0 1 | Total

-----------+----------------------+----------

0 | 69 338 | 407

| 76.67 59.30 | 61.67

-----------+----------------------+----------

1 | 21 232 | 253

| 23.33 40.70 | 38.33

-----------+----------------------+----------

Total | 90 570 | 660

| 100.00 100.00 | 100.00

Pearson chi2(1) = 9.9190 Pr = 0.002

ZINCDOSE_6 | ZINCDOSE_59MOS

MOS | 0 1 | Total

-----------+----------------------+----------

0 | 321 109 | 430

| 94.41 34.06 | 65.15

-----------+----------------------+----------

1 | 19 211 | 230

| 5.59 65.94 | 34.85

-----------+----------------------+----------

Total | 340 320 | 660

| 100.00 100.00 | 100.00

Pearson chi2(1) = 264.4360 Pr = 0.000

ZINCDOSE_6 | ZINCDURATION

MOS | 0 1 | Total

-----------+----------------------+----------

0 | 87 343 | 430

| 96.67 60.18 | 65.15

-----------+----------------------+----------

1 | 3 227 | 230

| 3.33 39.82 | 34.85

-----------+----------------------+----------

Total | 90 570 | 660

| 100.00 100.00 | 100.00

Pearson chi2(1) = 45.5871 Pr = 0.000

ZINCDOSE_5 | ZINCDURATION

9MOS | 0 1 | Total

-----------+----------------------+----------

0 | 83 257 | 340

| 92.22 45.09 | 51.52

-----------+----------------------+----------

1 | 7 313 | 320

| 7.78 54.91 | 48.48

-----------+----------------------+----------

Total | 90 570 | 660

| 100.00 100.00 | 100.00

Pearson chi2(1) = 69.1370 Pr = 0.000

| ZINCORS2

ZINCORS1 | 0 1 | Total

-----------+----------------------+----------

0 | 81 11 | 92

| 17.76 5.39 | 13.94

-----------+----------------------+----------

1 | 375 193 | 568

| 82.24 94.61 | 86.06

-----------+----------------------+----------

Total | 456 204 | 660

| 100.00 100.00 | 100.00

Pearson chi2(1) = 17.9809 Pr = 0.000

| ZINCORS3

ZINCORS1 | 0 1 | Total

-----------+----------------------+----------

0 | 69 23 | 92

| 27.82 5.58 | 13.94

-----------+----------------------+----------

1 | 179 389 | 568

| 72.18 94.42 | 86.06

-----------+----------------------+----------

Total | 248 412 | 660

| 100.00 100.00 | 100.00

Pearson chi2(1) = 63.8305 Pr = 0.000

| ZINCORS4

ZINCORS1 | 0 1 | Total

-----------+----------------------+----------

0 | 77 15 | 92

| 18.83 5.98 | 13.94

-----------+----------------------+----------

1 | 332 236 | 568

| 81.17 94.02 | 86.06

-----------+----------------------+----------

Total | 409 251 | 660

| 100.00 100.00 | 100.00

Pearson chi2(1) = 21.4108 Pr = 0.000

| ZINCDOSE_6MOS

ZINCORS1 | 0 1 | Total

-----------+----------------------+----------

0 | 76 16 | 92

| 17.67 6.96 | 13.94

-----------+----------------------+----------

1 | 354 214 | 568

| 82.33 93.04 | 86.06

-----------+----------------------+----------

Total | 430 230 | 660

| 100.00 100.00 | 100.00

Pearson chi2(1) = 14.3491 Pr = 0.000

| ZINCDOSE_59MOS

ZINCORS1 | 0 1 | Total

-----------+----------------------+----------

0 | 67 25 | 92

| 19.71 7.81 | 13.94

-----------+----------------------+----------

1 | 273 295 | 568

| 80.29 92.19 | 86.06

-----------+----------------------+----------

Total | 340 320 | 660

| 100.00 100.00 | 100.00

Pearson chi2(1) = 19.4378 Pr = 0.000

| ZINCDURATION

ZINCORS1 | 0 1 | Total

-----------+----------------------+----------

0 | 48 44 | 92

| 53.33 7.72 | 13.94

-----------+----------------------+----------

1 | 42 526 | 568

| 46.67 92.28 | 86.06

-----------+----------------------+----------

Total | 90 570 | 660

| 100.00 100.00 | 100.00

Pearson chi2(1) = 134.8100 Pr = 0.000

| ORSPREP

ZINCORS1 | 0 1 | Total

-----------+----------------------+----------

0 | 15 77 | 92

| 46.88 12.26 | 13.94

-----------+----------------------+----------

1 | 17 551 | 568

| 53.13 87.74 | 86.06

-----------+----------------------+----------

Total | 32 628 | 660

| 100.00 100.00 | 100.00

Pearson chi2(1) = 30.4101 Pr = 0.000

| ZINCORS3

ZINCORS2 | 0 1 | Total

-----------+----------------------+----------

0 | 205 251 | 456

| 82.66 60.92 | 69.09

-----------+----------------------+----------

1 | 43 161 | 204

| 17.34 39.08 | 30.91

-----------+----------------------+----------

Total | 248 412 | 660

| 100.00 100.00 | 100.00

Pearson chi2(1) = 34.2591 Pr = 0.000

| ZINCORS4

ZINCORS2 | 0 1 | Total

-----------+----------------------+----------

0 | 314 142 | 456

| 76.77 56.57 | 69.09

-----------+----------------------+----------

1 | 95 109 | 204

| 23.23 43.43 | 30.91

-----------+----------------------+----------

Total | 409 251 | 660

| 100.00 100.00 | 100.00

Pearson chi2(1) = 29.7168 Pr = 0.000

| ZINCDOSE_6MOS

ZINCORS2 | 0 1 | Total

-----------+----------------------+----------

0 | 302 154 | 456

| 70.23 66.96 | 69.09

-----------+----------------------+----------

1 | 128 76 | 204

| 29.77 33.04 | 30.91

-----------+----------------------+----------

Total | 430 230 | 660

| 100.00 100.00 | 100.00

Pearson chi2(1) = 0.7531 Pr = 0.386

| ZINCDOSE_59MOS

ZINCORS2 | 0 1 | Total

-----------+----------------------+----------

0 | 229 227 | 456

| 67.35 70.94 | 69.09

-----------+----------------------+----------

1 | 111 93 | 204

| 32.65 29.06 | 30.91

-----------+----------------------+----------

Total | 340 320 | 660

| 100.00 100.00 | 100.00

Pearson chi2(1) = 0.9919 Pr = 0.319

| ZINCDURATION

ZINCORS2 | 0 1 | Total

-----------+----------------------+----------

0 | 79 377 | 456

| 87.78 66.14 | 69.09

-----------+----------------------+----------

1 | 11 193 | 204

| 12.22 33.86 | 30.91

-----------+----------------------+----------

Total | 90 570 | 660

| 100.00 100.00 | 100.00

Pearson chi2(1) = 17.0403 Pr = 0.000

| ORSPREP

ZINCORS2 | 0 1 | Total

-----------+----------------------+----------

0 | 29 427 | 456

| 90.63 67.99 | 69.09

-----------+----------------------+----------

1 | 3 201 | 204

| 9.38 32.01 | 30.91

-----------+----------------------+----------

Total | 32 628 | 660

| 100.00 100.00 | 100.00

Pearson chi2(1) = 7.3026 Pr = 0.007

| ZINCORS4

ZINCORS3 | 0 1 | Total

-----------+----------------------+----------

0 | 209 39 | 248

| 51.10 15.54 | 37.58

-----------+----------------------+----------

1 | 200 212 | 412

| 48.90 84.46 | 62.42

-----------+----------------------+----------

Total | 409 251 | 660

| 100.00 100.00 | 100.00

Pearson chi2(1) = 83.8637 Pr = 0.000

| ZINCDOSE_6MOS

ZINCORS3 | 0 1 | Total

-----------+----------------------+----------

0 | 178 70 | 248

| 41.40 30.43 | 37.58

-----------+----------------------+----------

1 | 252 160 | 412

| 58.60 69.57 | 62.42

-----------+----------------------+----------

Total | 430 230 | 660

| 100.00 100.00 | 100.00

Pearson chi2(1) = 7.6746 Pr = 0.006

| ZINCDOSE_59MOS

ZINCORS3 | 0 1 | Total

-----------+----------------------+----------

0 | 135 113 | 248

| 39.71 35.31 | 37.58

-----------+----------------------+----------

1 | 205 207 | 412

| 60.29 64.69 | 62.42

-----------+----------------------+----------

Total | 340 320 | 660

| 100.00 100.00 | 100.00

Pearson chi2(1) = 1.3565 Pr = 0.244

| ZINCDURATION

ZINCORS3 | 0 1 | Total

-----------+----------------------+----------

0 | 57 191 | 248

| 63.33 33.51 | 37.58

-----------+----------------------+----------

1 | 33 379 | 412

| 36.67 66.49 | 62.42

-----------+----------------------+----------

Total | 90 570 | 660

| 100.00 100.00 | 100.00

Pearson chi2(1) = 29.4755 Pr = 0.000

| ORSPREP

ZINCORS3 | 0 1 | Total

-----------+----------------------+----------

0 | 18 230 | 248

| 56.25 36.62 | 37.58

-----------+----------------------+----------

1 | 14 398 | 412

| 43.75 63.38 | 62.42

-----------+----------------------+----------

Total | 32 628 | 660

| 100.00 100.00 | 100.00

Pearson chi2(1) = 4.9999 Pr = 0.025

| ZINCDOSE_6MOS

ZINCORS4 | 0 1 | Total

-----------+----------------------+----------

0 | 268 141 | 409

| 62.33 61.30 | 61.97

-----------+----------------------+----------

1 | 162 89 | 251

| 37.67 38.70 | 38.03

-----------+----------------------+----------

Total | 430 230 | 660

| 100.00 100.00 | 100.00

Pearson chi2(1) = 0.0663 Pr = 0.797

| ZINCDOSE_59MOS

ZINCORS4 | 0 1 | Total

-----------+----------------------+----------

0 | 213 196 | 409

| 62.65 61.25 | 61.97

-----------+----------------------+----------

1 | 127 124 | 251

| 37.35 38.75 | 38.03

-----------+----------------------+----------

Total | 340 320 | 660

| 100.00 100.00 | 100.00

Pearson chi2(1) = 0.1365 Pr = 0.712

| ZINCDURATION

ZINCORS4 | 0 1 | Total

-----------+----------------------+----------

0 | 69 340 | 409

| 76.67 59.65 | 61.97

-----------+----------------------+----------

1 | 21 230 | 251

| 23.33 40.35 | 38.03

-----------+----------------------+----------

Total | 90 570 | 660

| 100.00 100.00 | 100.00

Pearson chi2(1) = 9.5512 Pr = 0.002

| ORSPREP

ZINCORS4 | 0 1 | Total

-----------+----------------------+----------

0 | 24 385 | 409

| 75.00 61.31 | 61.97

-----------+----------------------+----------

1 | 8 243 | 251

| 25.00 38.69 | 38.03

-----------+----------------------+----------

Total | 32 628 | 660

| 100.00 100.00 | 100.00

Pearson chi2(1) = 2.4229 Pr = 0.120

S1 Appendix C. Contingency Tables – Private Sector

| ORS2

ORS1 | 0 1 | Total

-----------+----------------------+----------

0 | 13 8 | 21

| 29.55 15.09 | 21.65

-----------+----------------------+----------

1 | 31 45 | 76

| 70.45 84.91 | 78.35

-----------+----------------------+----------

Total | 44 53 | 97

| 100.00 100.00 | 100.00

Pearson chi2(1) = 2.9599 Pr = 0.085

| ORS3

ORS1 | 0 1 | Total

-----------+----------------------+----------

0 | 13 8 | 21

| 23.64 19.05 | 21.65

-----------+----------------------+----------

1 | 42 34 | 76

| 76.36 80.95 | 78.35

-----------+----------------------+----------

Total | 55 42 | 97

| 100.00 100.00 | 100.00

Pearson chi2(1) = 0.2956 Pr = 0.587

| ORS4

ORS1 | 0 1 | Total

-----------+----------------------+----------

0 | 16 5 | 21

| 30.19 11.36 | 21.65

-----------+----------------------+----------

1 | 37 39 | 76

| 69.81 88.64 | 78.35

-----------+----------------------+----------

Total | 53 44 | 97

| 100.00 100.00 | 100.00

Pearson chi2(1) = 5.0227 Pr = 0.025

| ORSPREP

ORS1 | 0 1 | Total

-----------+----------------------+----------

0 | 10 11 | 21

| 21.28 22.00 | 21.65

-----------+----------------------+----------

1 | 37 39 | 76

| 78.72 78.00 | 78.35

-----------+----------------------+----------

Total | 47 50 | 97

| 100.00 100.00 | 100.00

Pearson chi2(1) = 0.0075 Pr = 0.931

| ORS3

ORS2 | 0 1 | Total

-----------+----------------------+----------

0 | 35 9 | 44

| 63.64 21.43 | 45.36

-----------+----------------------+----------

1 | 20 33 | 53

| 36.36 78.57 | 54.64

-----------+----------------------+----------

Total | 55 42 | 97

| 100.00 100.00 | 100.00

Pearson chi2(1) = 17.1175 Pr = 0.000

| ORS4

ORS2 | 0 1 | Total

-----------+----------------------+----------

0 | 29 15 | 44

| 54.72 34.09 | 45.36

-----------+----------------------+----------

1 | 24 29 | 53

| 45.28 65.91 | 54.64

-----------+----------------------+----------

Total | 53 44 | 97

| 100.00 100.00 | 100.00

Pearson chi2(1) = 4.1267 Pr = 0.042

| ORSPREP

ORS2 | 0 1 | Total

-----------+----------------------+----------

0 | 22 22 | 44

| 46.81 44.00 | 45.36

-----------+----------------------+----------

1 | 25 28 | 53

| 53.19 56.00 | 54.64

-----------+----------------------+----------

Total | 47 50 | 97

| 100.00 100.00 | 100.00

Pearson chi2(1) = 0.0771 Pr = 0.781

| ORS4

ORS3 | 0 1 | Total

-----------+----------------------+----------

0 | 35 20 | 55

| 66.04 45.45 | 56.70

-----------+----------------------+----------

1 | 18 24 | 42

| 33.96 54.55 | 43.30

-----------+----------------------+----------

Total | 53 44 | 97

| 100.00 100.00 | 100.00

Pearson chi2(1) = 4.1487 Pr = 0.042

| ORSPREP

ORS3 | 0 1 | Total

-----------+----------------------+----------

0 | 31 24 | 55

| 65.96 48.00 | 56.70

-----------+----------------------+----------

1 | 16 26 | 42

| 34.04 52.00 | 43.30

-----------+----------------------+----------

Total | 47 50 | 97

| 100.00 100.00 | 100.00

Pearson chi2(1) = 3.1821 Pr = 0.074

| ORSPREP

ORS4 | 0 1 | Total

-----------+----------------------+----------

0 | 23 30 | 53

| 48.94 60.00 | 54.64

-----------+----------------------+----------

1 | 24 20 | 44

| 51.06 40.00 | 45.36

-----------+----------------------+----------

Total | 47 50 | 97

| 100.00 100.00 | 100.00

Pearson chi2(1) = 1.1965 Pr = 0.274

| ZINC2

ZINC1 | 0 1 | Total

-----------+----------------------+----------

0 | 50 9 | 59

| 74.63 30.00 | 60.82

-----------+----------------------+----------

1 | 17 21 | 38

| 25.37 70.00 | 39.18

-----------+----------------------+----------

Total | 67 30 | 97

| 100.00 100.00 | 100.00

Pearson chi2(1) = 17.3191 Pr = 0.000

| ZINC3

ZINC1 | 0 1 | Total

-----------+----------------------+----------

0 | 50 9 | 59

| 70.42 34.62 | 60.82

-----------+----------------------+----------

1 | 21 17 | 38

| 29.58 65.38 | 39.18

-----------+----------------------+----------

Total | 71 26 | 97

| 100.00 100.00 | 100.00

Pearson chi2(1) = 10.2402 Pr = 0.001

| ZINC4

ZINC1 | 0 1 | Total

-----------+----------------------+----------

0 | 53 6 | 59

| 71.62 26.09 | 60.82

-----------+----------------------+----------

1 | 21 17 | 38

| 28.38 73.91 | 39.18

-----------+----------------------+----------

Total | 74 23 | 97

| 100.00 100.00 | 100.00

Pearson chi2(1) = 15.2679 Pr = 0.000

| ZINCDOSE_6MOS

ZINC1 | 0 1 | Total

-----------+----------------------+----------

0 | 56 3 | 59

| 61.54 50.00 | 60.82

-----------+----------------------+----------

1 | 35 3 | 38

| 38.46 50.00 | 39.18

-----------+----------------------+----------

Total | 91 6 | 97

| 100.00 100.00 | 100.00

Pearson chi2(1) = 0.3145 Pr = 0.575

| ZINCDOSE_59MOS

ZINC1 | 0 1 | Total

-----------+----------------------+----------

0 | 54 5 | 59

| 59.34 83.33 | 60.82

-----------+----------------------+----------

1 | 37 1 | 38

| 40.66 16.67 | 39.18

-----------+----------------------+----------

Total | 91 6 | 97

| 100.00 100.00 | 100.00

Pearson chi2(1) = 1.3598 Pr = 0.244

| ZINCDURATION

ZINC1 | 0 1 | Total

-----------+----------------------+----------

0 | 29 30 | 59

| 64.44 57.69 | 60.82

-----------+----------------------+----------

1 | 16 22 | 38

| 35.56 42.31 | 39.18

-----------+----------------------+----------

Total | 45 52 | 97

| 100.00 100.00 | 100.00

Pearson chi2(1) = 0.4616 Pr = 0.497

| ZINC3

ZINC2 | 0 1 | Total

-----------+----------------------+----------

0 | 56 11 | 67

| 78.87 42.31 | 69.07

-----------+----------------------+----------

1 | 15 15 | 30

| 21.13 57.69 | 30.93

-----------+----------------------+----------

Total | 71 26 | 97

| 100.00 100.00 | 100.00

Pearson chi2(1) = 11.9111 Pr = 0.001

| ZINC4

ZINC2 | 0 1 | Total

-----------+----------------------+----------

0 | 60 7 | 67

| 81.08 30.43 | 69.07

-----------+----------------------+----------

1 | 14 16 | 30

| 18.92 69.57 | 30.93

-----------+----------------------+----------

Total | 74 23 | 97

| 100.00 100.00 | 100.00

Pearson chi2(1) = 21.0684 Pr = 0.000

| ZINCDOSE_6MOS

ZINC2 | 0 1 | Total

-----------+----------------------+----------

0 | 63 4 | 67

| 69.23 66.67 | 69.07

-----------+----------------------+----------

1 | 28 2 | 30

| 30.77 33.33 | 30.93

-----------+----------------------+----------

Total | 91 6 | 97

| 100.00 100.00 | 100.00

Pearson chi2(1) = 0.0173 Pr = 0.895

| ZINCDOSE_59MOS

ZINC2 | 0 1 | Total

-----------+----------------------+----------

0 | 64 3 | 67

| 70.33 50.00 | 69.07

-----------+----------------------+----------

1 | 27 3 | 30

| 29.67 50.00 | 30.93

-----------+----------------------+----------

Total | 91 6 | 97

| 100.00 100.00 | 100.00

Pearson chi2(1) = 1.0890 Pr = 0.297

| ZINCDURATION

ZINC2 | 0 1 | Total

-----------+----------------------+----------

0 | 37 30 | 67

| 82.22 57.69 | 69.07

-----------+----------------------+----------

1 | 8 22 | 30

| 17.78 42.31 | 30.93

-----------+----------------------+----------

Total | 45 52 | 97

| 100.00 100.00 | 100.00

Pearson chi2(1) = 6.7949 Pr = 0.009

| ZINC4

ZINC3 | 0 1 | Total

-----------+----------------------+----------

0 | 62 9 | 71

| 83.78 39.13 | 73.20

-----------+----------------------+----------

1 | 12 14 | 26

| 16.22 60.87 | 26.80

-----------+----------------------+----------

Total | 74 23 | 97

| 100.00 100.00 | 100.00

Pearson chi2(1) = 17.8323 Pr = 0.000

| ZINCDOSE_6MOS

ZINC3 | 0 1 | Total

-----------+----------------------+----------

0 | 67 4 | 71

| 73.63 66.67 | 73.20

-----------+----------------------+----------

1 | 24 2 | 26

| 26.37 33.33 | 26.80

-----------+----------------------+----------

Total | 91 6 | 97

| 100.00 100.00 | 100.00

Pearson chi2(1) = 0.1390 Pr = 0.709

| ZINCDOSE_59MOS

ZINC3 | 0 1 | Total

-----------+----------------------+----------

0 | 67 4 | 71

| 73.63 66.67 | 73.20

-----------+----------------------+----------

1 | 24 2 | 26

| 26.37 33.33 | 26.80

-----------+----------------------+----------

Total | 91 6 | 97

| 100.00 100.00 | 100.00

Pearson chi2(1) = 0.1390 Pr = 0.709

| ZINCDURATION

ZINC3 | 0 1 | Total

-----------+----------------------+----------

0 | 35 36 | 71

| 77.78 69.23 | 73.20

-----------+----------------------+----------

1 | 10 16 | 26

| 22.22 30.77 | 26.80

-----------+----------------------+----------

Total | 45 52 | 97

| 100.00 100.00 | 100.00

Pearson chi2(1) = 0.8982 Pr = 0.343

| ZINCDOSE_6MOS

ZINC4 | 0 1 | Total

-----------+----------------------+----------

0 | 69 5 | 74

| 75.82 83.33 | 76.29

-----------+----------------------+----------

1 | 22 1 | 23

| 24.18 16.67 | 23.71

-----------+----------------------+----------

Total | 91 6 | 97

| 100.00 100.00 | 100.00

Pearson chi2(1) = 0.1755 Pr = 0.675

| ZINCDOSE_59MOS

ZINC4 | 0 1 | Total

-----------+----------------------+----------

0 | 69 5 | 74

| 75.82 83.33 | 76.29

-----------+----------------------+----------

1 | 22 1 | 23

| 24.18 16.67 | 23.71

-----------+----------------------+----------

Total | 91 6 | 97

| 100.00 100.00 | 100.00

Pearson chi2(1) = 0.1755 Pr = 0.675

| ZINCDURATION

ZINC4 | 0 1 | Total

-----------+----------------------+----------

0 | 38 36 | 74

| 84.44 69.23 | 76.29

-----------+----------------------+----------

1 | 7 16 | 23

| 15.56 30.77 | 23.71

-----------+----------------------+----------

Total | 45 52 | 97

| 100.00 100.00 | 100.00

Pearson chi2(1) = 3.0867 Pr = 0.079

ZINCDOSE_6 | ZINCDOSE_59MOS

MOS | 0 1 | Total

-----------+----------------------+----------

0 | 89 2 | 91

| 97.80 33.33 | 93.81

-----------+----------------------+----------

1 | 2 4 | 6

| 2.20 66.67 | 6.19

-----------+----------------------+----------

Total | 91 6 | 97

| 100.00 100.00 | 100.00

Pearson chi2(1) = 40.3155 Pr = 0.000

ZINCDOSE_6 | ZINCDURATION

MOS | 0 1 | Total

-----------+----------------------+----------

0 | 44 47 | 91

| 97.78 90.38 | 93.81

-----------+----------------------+----------

1 | 1 5 | 6

| 2.22 9.62 | 6.19

-----------+----------------------+----------

Total | 45 52 | 97

| 100.00 100.00 | 100.00

Pearson chi2(1) = 2.2722 Pr = 0.132

ZINCDOSE_5 | ZINCDURATION

9MOS | 0 1 | Total

-----------+----------------------+----------

0 | 43 48 | 91

| 95.56 92.31 | 93.81

-----------+----------------------+----------

1 | 2 4 | 6

| 4.44 7.69 | 6.19

-----------+----------------------+----------

Total | 45 52 | 97

| 100.00 100.00 | 100.00

Pearson chi2(1) = 0.4385 Pr = 0.508

| ZINCORS2

ZINCORS1 | 0 1 | Total

-----------+----------------------+----------

0 | 53 9 | 62

| 76.81 32.14 | 63.92

-----------+----------------------+----------

1 | 16 19 | 35

| 23.19 67.86 | 36.08

-----------+----------------------+----------

Total | 69 28 | 97

| 100.00 100.00 | 100.00

Pearson chi2(1) = 17.2316 Pr = 0.000

| ZINCORS3

ZINCORS1 | 0 1 | Total

-----------+----------------------+----------

0 | 52 10 | 62

| 72.22 40.00 | 63.92

-----------+----------------------+----------

1 | 20 15 | 35

| 27.78 60.00 | 36.08

-----------+----------------------+----------

Total | 72 25 | 97

| 100.00 100.00 | 100.00

Pearson chi2(1) = 8.3540 Pr = 0.004

| ZINCORS4

ZINCORS1 | 0 1 | Total

-----------+----------------------+----------

0 | 58 4 | 62

| 70.73 26.67 | 63.92

-----------+----------------------+----------

1 | 24 11 | 35

| 29.27 73.33 | 36.08

-----------+----------------------+----------

Total | 82 15 | 97

| 100.00 100.00 | 100.00

Pearson chi2(1) = 10.6759 Pr = 0.001

| ZINCDOSE_6MOS

ZINCORS1 | 0 1 | Total

-----------+----------------------+----------

0 | 59 3 | 62

| 64.84 50.00 | 63.92

-----------+----------------------+----------

1 | 32 3 | 35

| 35.16 50.00 | 36.08

-----------+----------------------+----------

Total | 91 6 | 97

| 100.00 100.00 | 100.00

Pearson chi2(1) = 0.5371 Pr = 0.464

| ZINCDOSE_59MOS

ZINCORS1 | 0 1 | Total

-----------+----------------------+----------

0 | 57 5 | 62

| 62.64 83.33 | 63.92

-----------+----------------------+----------

1 | 34 1 | 35

| 37.36 16.67 | 36.08

-----------+----------------------+----------

Total | 91 6 | 97

| 100.00 100.00 | 100.00

Pearson chi2(1) = 1.0454 Pr = 0.307

| ZINCDURATION

ZINCORS1 | 0 1 | Total

-----------+----------------------+----------

0 | 29 33 | 62

| 64.44 63.46 | 63.92

-----------+----------------------+----------

1 | 16 19 | 35

| 35.56 36.54 | 36.08

-----------+----------------------+----------

Total | 45 52 | 97

| 100.00 100.00 | 100.00

Pearson chi2(1) = 0.0101 Pr = 0.920

| ORSPREP

ZINCORS1 | 0 1 | Total

-----------+----------------------+----------

0 | 30 32 | 62

| 63.83 64.00 | 63.92

-----------+----------------------+----------

1 | 17 18 | 35

| 36.17 36.00 | 36.08

-----------+----------------------+----------

Total | 47 50 | 97

| 100.00 100.00 | 100.00

Pearson chi2(1) = 0.0003 Pr = 0.986

| ZINCORS3

ZINCORS2 | 0 1 | Total

-----------+----------------------+----------

0 | 58 11 | 69

| 80.56 44.00 | 71.13

-----------+----------------------+----------

1 | 14 14 | 28

| 19.44 56.00 | 28.87

-----------+----------------------+----------

Total | 72 25 | 97

| 100.00 100.00 | 100.00

Pearson chi2(1) = 12.0766 Pr = 0.001

| ZINCORS4

ZINCORS2 | 0 1 | Total

-----------+----------------------+----------

0 | 64 5 | 69

| 78.05 33.33 | 71.13

-----------+----------------------+----------

1 | 18 10 | 28

| 21.95 66.67 | 28.87

-----------+----------------------+----------

Total | 82 15 | 97

| 100.00 100.00 | 100.00

Pearson chi2(1) = 12.3477 Pr = 0.000

| ZINCDOSE_6MOS

ZINCORS2 | 0 1 | Total

-----------+----------------------+----------

0 | 65 4 | 69

| 71.43 66.67 | 71.13

-----------+----------------------+----------

1 | 26 2 | 28

| 28.57 33.33 | 28.87

-----------+----------------------+----------

Total | 91 6 | 97

| 100.00 100.00 | 100.00

Pearson chi2(1) = 0.0622 Pr = 0.803

| ZINCDOSE_59MOS

ZINCORS2 | 0 1 | Total

-----------+----------------------+----------

0 | 66 3 | 69

| 72.53 50.00 | 71.13

-----------+----------------------+----------

1 | 25 3 | 28

| 27.47 50.00 | 28.87

-----------+----------------------+----------

Total | 91 6 | 97

| 100.00 100.00 | 100.00

Pearson chi2(1) = 1.3912 Pr = 0.238

| ZINCDURATION

ZINCORS2 | 0 1 | Total

-----------+----------------------+----------

0 | 37 32 | 69

| 82.22 61.54 | 71.13

-----------+----------------------+----------

1 | 8 20 | 28

| 17.78 38.46 | 28.87

-----------+----------------------+----------

Total | 45 52 | 97

| 100.00 100.00 | 100.00

Pearson chi2(1) = 5.0262 Pr = 0.025

| ORSPREP

ZINCORS2 | 0 1 | Total

-----------+----------------------+----------

0 | 35 34 | 69

| 74.47 68.00 | 71.13

-----------+----------------------+----------

1 | 12 16 | 28

| 25.53 32.00 | 28.87

-----------+----------------------+----------

Total | 47 50 | 97

| 100.00 100.00 | 100.00

Pearson chi2(1) = 0.4936 Pr = 0.482

| ZINCORS4

ZINCORS3 | 0 1 | Total

-----------+----------------------+----------

0 | 68 4 | 72

| 82.93 26.67 | 74.23

-----------+----------------------+----------

1 | 14 11 | 25

| 17.07 73.33 | 25.77

-----------+----------------------+----------

Total | 82 15 | 97

| 100.00 100.00 | 100.00

Pearson chi2(1) = 20.9800 Pr = 0.000

| ZINCDOSE_6MOS

ZINCORS3 | 0 1 | Total

-----------+----------------------+----------

0 | 68 4 | 72

| 74.73 66.67 | 74.23

-----------+----------------------+----------

1 | 23 2 | 25

| 25.27 33.33 | 25.77

-----------+----------------------+----------

Total | 91 6 | 97

| 100.00 100.00 | 100.00

Pearson chi2(1) = 0.1911 Pr = 0.662

| ZINCDOSE_59MOS

ZINCORS3 | 0 1 | Total

-----------+----------------------+----------

0 | 68 4 | 72

| 74.73 66.67 | 74.23

-----------+----------------------+----------

1 | 23 2 | 25

| 25.27 33.33 | 25.77

-----------+----------------------+----------

Total | 91 6 | 97

| 100.00 100.00 | 100.00

Pearson chi2(1) = 0.1911 Pr = 0.662

| ZINCDURATION

ZINCORS3 | 0 1 | Total

-----------+----------------------+----------

0 | 35 37 | 72

| 77.78 71.15 | 74.23

-----------+----------------------+----------

1 | 10 15 | 25

| 22.22 28.85 | 25.77

-----------+----------------------+----------

Total | 45 52 | 97

| 100.00 100.00 | 100.00

Pearson chi2(1) = 0.5533 Pr = 0.457

| ORSPREP

ZINCORS3 | 0 1 | Total

-----------+----------------------+----------

0 | 37 35 | 72

| 78.72 70.00 | 74.23

-----------+----------------------+----------

1 | 10 15 | 25

| 21.28 30.00 | 25.77

-----------+----------------------+----------

Total | 47 50 | 97

| 100.00 100.00 | 100.00

Pearson chi2(1) = 0.9637 Pr = 0.326

| ZINCDOSE_6MOS

ZINCORS4 | 0 1 | Total

-----------+----------------------+----------

0 | 77 5 | 82

| 84.62 83.33 | 84.54

-----------+----------------------+----------

1 | 14 1 | 15

| 15.38 16.67 | 15.46

-----------+----------------------+----------

Total | 91 6 | 97

| 100.00 100.00 | 100.00

Pearson chi2(1) = 0.0071 Pr = 0.933

| ZINCDOSE_59MOS

ZINCORS4 | 0 1 | Total

-----------+----------------------+----------

0 | 77 5 | 82

| 84.62 83.33 | 84.54

-----------+----------------------+----------

1 | 14 1 | 15

| 15.38 16.67 | 15.46

-----------+----------------------+----------

Total | 91 6 | 97

| 100.00 100.00 | 100.00

Pearson chi2(1) = 0.0071 Pr = 0.933

| ZINCDURATION

ZINCORS4 | 0 1 | Total

-----------+----------------------+----------

0 | 41 41 | 82

| 91.11 78.85 | 84.54

-----------+----------------------+----------

1 | 4 11 | 15

| 8.89 21.15 | 15.46

-----------+----------------------+----------

Total | 45 52 | 97

| 100.00 100.00 | 100.00

Pearson chi2(1) = 2.7760 Pr = 0.096

| ORSPREP

ZINCORS4 | 0 1 | Total

-----------+----------------------+----------

0 | 39 43 | 82

| 82.98 86.00 | 84.54

-----------+----------------------+----------

1 | 8 7 | 15

| 17.02 14.00 | 15.46

-----------+----------------------+----------

Total | 47 50 | 97

| 100.00 100.00 | 100.00

Pearson chi2(1) = 0.1692 Pr = 0.681
